# Being psychotic is not necessarily being ill: Psychotic continuum and the relevance of lacanian psychoanalytic approach

**DOI:** 10.1192/j.eurpsy.2021.2127

**Published:** 2021-08-13

**Authors:** G. Mitropoulos

**Affiliations:** 9th Department, Psychiatric Hospital of Attika, Athens, Greece

**Keywords:** psychosis, lacanian psychoanalysis, continuum, coping

## Abstract

**Introduction:**

The notion of subclinical psychosis is as old as Eugen Bleuler’s work on schizophrenia. It is also consistent with psychodynamic theories (see PDM-2) on the organization of personality on different levels including, among others, a psychotic level of personality organization. Research on the continuum of psychosis has offered substantial support to the view that psychotic phenotypes are significantly more prevalent than clinical psychosis.

**Objectives:**

This may imply that being “psychotic” is not necessarily being ill. This assumption raises important theoretical and clinical questions: what causes psychosis to manifest itself clinically and, conversely, what possibly prevents it from doing so?

**Methods:**

At the same time, it potentially frees psychiatry from certain diagnostic and therapeutic impasses. It allows for a shift of emphasis from misguiding classifications and often frustrating “evidence-based” therapeutic attempts to a more personalized approach.

**Results:**

Diagnosis may thus rely on psychoanalytical “markers” or “indicators” regarding the subject (e.g. deficits in the symbolic register, dysregulated rapport with one’s body, problematic inscription in social relations etc.) at least as much as on symptomic phenomenology. Therapy may also take advantage of and deploy the unique coping strategies employed by the psychotic individual.

**Conclusions:**

The diagnostic and therapeutic insights offered by Lacanian psychoanalysis create the possibility of a fruitful theoretical, diagnostic and therapeutic approach for clinical and subclinical psychotic conditions; indicate that psychoanalysis is indispensable for clinical psychiatry; and signal the possibility of a time-honored alternative to the in-vogue neurocognitive paradigm of “personalized” psychiatry.

**Disclosure:**

No significant relationships.

